# Field-captured *Aedes vexans* (Meigen, 1830) is a competent vector for Rift Valley fever phlebovirus in Europe

**DOI:** 10.1186/s13071-019-3728-9

**Published:** 2019-10-16

**Authors:** Lotty Birnberg, Sandra Talavera, Carles Aranda, Ana I. Núñez, Sebastian Napp, Núria Busquets

**Affiliations:** 1 Centre de Recerca en Sanitat Animal (CReSA), Institut de Recerca en Tecnologies Agroalimentaries (IRTA), Barcelona, Spain; 2 Servei de Control de Mosquits del Consell Comarcal del Baix Llobregat, Barcelona, Spain

**Keywords:** *Aedes vexans*, RVFV, Mosquito Flavivirus, Vector competence

## Abstract

**Background:**

*Aedes vexans* (Meigen) is considered a nuisance species in central Europe and the Mediterranean region. It is an anthropophilic and mammalophilic floodwater mosquito involved in the transmission of several arboviruses. Rift Valley fever (RVF) is a relevant mosquito-borne zoonosis, affecting mainly humans and ruminants, that causes severe impact in public health and economic loses. Due to globalization and climate change, the European continent is threatened by its introduction. The main purpose of the present study was to evaluate the vector competence of a European field-collected *Ae. vexans* population.

**Methods:**

*Aedes vexans* field-collected larvae were reared in the laboratory under field-simulated conditions. To assess the vector competence for Rift Valley fever phlebovirus (RVFV) transmission, adult F0 females were exposed to infectious blood meals containing the 56/74 RVFV strain. Additionally, intrathoracic inoculations with the same virus strain were performed to evaluate the relevance of the salivary gland barriers. Natural circulation of alphavirus, flavivirus and phlebovirus was also tested.

**Results:**

To our knowledge, an autochthonous *Ae. vexans* population was experimentally confirmed as a competent vector for RVFV for the first time. This virus was capable of infecting and disseminating within the studied *Ae. vexans* mosquitoes. Moreover, infectious virus was isolated from the saliva of disseminated specimens, showing their capacity to transmit the virus. Additionally, a natural infection with a circulating Mosquito flavivirus was detected. The co-infection with the Mosquito flavivirus seemed to modulate RVFV infection susceptibility in field-collected *Ae. vexans*, but further studies are needed to confirm its potential interference in RVFV transmission.

**Conclusions:**

Our results show that field-collected European *Ae. vexans* would be able to transmit RVFV in case of introduction into the continent. This should be taken into consideration in the design of surveillance and control programmes.

## Background

*Aedes vexans* (Meigen, 1830) is a floodwater mosquito widely distributed throughout the Holarctic region and it is native in eastern Europe. This species inhabits a variety of habitats, especially within rural areas [1]. It mostly breeds in floodplains, rivers and lakes. As most floodwater mosquitoes, *Ae. vexans* lay their eggs near temporary or semi-permanent ground pools predisposed to seasonal inundations. Their eggs in diapause survive long periods of drought and hatch massively after flooding episodes. *Aedes vexans* is able to complete its developmental cycle in only a few days producing high population densities [2]. Adult females are aggressive bitters with low host specificity among mammals and humans [3], relevant for potential pathogen transmission. In North America and Europe, several arboviruses, such as West Nile virus (WNV), Snowshoe hare virus (SSHV), Jamestone Canyon virus (JCV) [4], Tahyna virus (TAHV) [5], and Batai virus (BATV) [6] to name a few, have been isolated from *Ae. vexans*. In Africa, *Ae. vexans* is considered one of the primary vectors of Rift Valley fever phlebovirus (RVFV) [7, 8], and has been found naturally infected with the virus [9]. In addition, its competence in the transmission of RVFV has been confirmed experimentally in field populations from Africa and the USA [9–11].

Rift Valley fever (RVF) is a zoonotic vector-borne viral disease that mainly affects domesticated ruminants and humans. Rift Valley fever is responsible for high mortality rates in newborn and juvenile ruminants, and abortions in pregnant animals [12]. Human infections may vary from an asymptomatic to mild febrile illness, but 1% of them may develop into severe encephalitis, haemorrhagic fever and death [13]. Its causal agent, RVFV, belongs to the genus *Phlebovirus* within the family *Phenuiviridae*. Unlike most phleboviruses, which are primarily transmitted by sand flies, RVFV is transmitted predominantly by infected mosquito bites [11].

Due to its dreadful impacts on public health and the economy in endemic countries, RVFV belongs to the World Organization for Animal Health (OIE)’s list of notifiable animal diseases of concern, and is classified as a category A priority pathogen by the National Institute of Allergy and Infectious Diseases (NIAID) [14]. In the last decades, RVFV distribution has expanded from its original location in sub-Saharan Africa to North and West Africa, the Arabian Peninsula, Mayotte Island and Madagascar [9, 12, 13, 15]. Although no RVF cases have been reported in Europe so far, globalization and climate change have raised concerns of its introduction through the Mediterranean basin. While predictive risk models of the introduction of RVF within the European Union have reported a low risk [12], a study using a spatial multicriteria decision-making (MCDM) model for RVF outbreak occurrence in Spain, showed a high suitability for RVF in the east-coast regions [16], where *Ae. vexans* mosquito is present.

For a better understanding of the potential role in the transmission of RVFV of an autochthonous population of *Ae. vexans* in Europe, we tested the ability of field-captured *Ae. vexans* mosquitoes from Begues municipality in Catalonia (Spain) for the transmission of RVFV.

## Methods

### Sample collection and mosquito rearing

In September 2016 and May 2019, after heavy rain episodes, *Ae. vexans* third- and fourth-instar larvae were collected from Begues municipality (41°19′57.94″N, 1°54′20.40″E) (Catalonia, Spain). To obtain an F0 generation, larvae were reared in the laboratory under local field-simulated conditions (photoperiod 14 h day:10 h night, relative humidity: 80%, temperature: 22–26 °C) using the same water and substrate from their original breeding site. Specimen identification was based on morphology as described by Schaffner et al. [17].

### Virus strain and inoculum preparation

A South African virulent 56/74 RVFV strain (viral stock provided by Alejandro Brun, INIA), isolated from cattle in 1974 [18] was used. The virus was passaged twice in *Aedes albopictus* clone C6/36 cells and titrated in African green monkey kidney (Vero) cells (both cell lines provided by Joan Pujols, IRTA-CReSA, Barcelona, Spain) to obtain a 50% tissue culture infective dose per millilitre (TCID_50_/ml) [19]. For mosquito blood meals, fresh heparinized bovine blood (Servei de granja i camps experimentals (SGICE), Veterinary Faculty, Autonomous University of Barcelona) was supplemented with adenosine 5’-triphosphate (ATP) disodium salt hydrate (5 × 10^−3^ M) (Sigma-Aldrich, St. Louis, MO, USA) as phagostimulant. Infectious blood meals were prepared by mixing (1:3) bovine blood and virus to obtain a final concentration of 7.5 log_10_ TCID_50_/ml. The viral dose employed in our assay was similar to those detected previously in blood samples from experimentally infected European lambs [19].

### Design of the vector competence assay

The competence for the transmission of RVFV of a European field-captured *Ae. vexans* population was assessed in two different years, 2016 and 2019. In 2016, at the Institut de Recerca i Tecnologies Agroalimentaries – Centre de Recerca en Sanitat Animal (IRTA – CReSA) Biosafety Level 3 (BSL3) facilities, 422 non-blood-fed F0 females aged 7–9 days were exposed to artificial feedings. All F0 females were starved for 24 h and exposed to an infectious blood meal that was performed using a Hemotek feeding system (Discovery Workshop, Accrington, UK) set at 37.5 °C for one hour. A specific pathogen-free (SPF) chicken skin served as a feeding membrane. Simultaneously, a virus-free blood meal was offered to one group to obtain a negative control. After feedings, specimens were anesthetized by exposing them to carbon dioxide (CO_2_); fully engorged females (FEF) were separated and kept in individual cardboard cages (Watkins & Doncaster, Leominster, UK) under rearing conditions (photoperiod 14 h day:10 h night, relative humidity: 80%, temperature: 22–26 °C). On the same day of feeding, three FEF from each group were sacrificed to verify the presence or absence of the virus. The remaining unfed females were maintained deprived of sucrose for another 24 h and subjected to a second feeding (following 48 h of starvation). The same procedure for feeding and female classification were performed, with the only difference to verify the infectious status, five FEF were sacrificed per group. Twenty-one (17 exposed to RVFV and four from the negative control) and 40 FEF (35 exposed to RVFV and five from the negative control), from the first and second feeding, respectively, were maintained for 14 days under rearing conditions until the completion of the extrinsic incubation period (EIP).

At 14 dpe, all specimens were anesthetized with CO_2_. Legs and wings were removed from the body of each specimen and stored in 1.5 ml tubes containing 0.5 ml Dulbecco’s modified Eagle’s medium (DMEM) (Lonza, Basel, Switzerland). Immediately after dissection, saliva samples were collected by the capillary technique used by Brustolin et al. [20]. All samples were stored at − 80 °C until processed. Specimens from the negative control group helped to verify the survival of the studied individuals and their infection status until the end of the experiment.

In 2019, 229 F0 females were obtained from field-collected larvae. Prior artificial feeding, 148 7–9 day-old non-blood-fed females were deprived of sucrose for 48 h to ensure a higher feeding rate. Artificial feeding, specimen maintenance, sample collection and processing were performed as described above for the previous assay.

Since the number of disseminated specimens after oral exposure to RVFV was low, to better evaluate the transmission rate of this mosquito population, as well as, to assess the relevance of the salivary glands barriers, intrathoracic inoculations were performed.

### RVFV intrathoracic inoculation in mosquitoes

Using a XenoWorks analog microinjector (BRI) (Sutter Instrument, CA, USA), 67 9–12 day-old females, from the same 2019 batch, were inoculated with 1–2 µl of the same RVFV strain (5.67 log_10_ TCID_50_) previously used in artificial feeding assays. Fourteen specimens were inoculated with sterile PBS as an inoculation and survival control. To confirm the infection status, five specimens were sacrificed the same day of microinjection. Inoculated specimens were maintained individually for 7 days under previous rearing conditions. At day 7 post-inoculation (7 dpi) all specimens were anesthetized with CO_2_, legs and wings were detached from the body, and the saliva of all females harvested as previously described for artificial feeding. Bodies, legs and wings, and saliva samples were stored at − 80 °C until molecular analysis could be completed.

### Detection and isolation of RVFV

Viral RNA was extracted from bodies, legs and wings, and saliva samples using NucleoSpin® RNA Virus kit (Macherey-Nagel, Düren, Germany). RVFV detection and quantification were performed following the protocol previously described [20] where the limit of detection was established at 0.09 TCID_50_ per reaction. Quantification cycle (Cq) values below 36 were considered positive for RVFV. Saliva samples were also incubated in Vero cells (37 °C, 5% CO_2_) for RVFV isolation for 7 days, before cytopathic effect was visually evaluated.

### Parameters to evaluate *Ae. vexans* vector competence for RVFV

At 14 dpe, infection, disseminated infection and transmission rates (IR, DIR and TR, respectively), and transmission efficiency (TE) were estimated. IR corresponds to the fraction of FEF whose bodies tested positive for RVFV. DIR is the proportion of FEF with RVFV infection in legs and wings among FEF with infected bodies. TR is the proportion of FEF with RVFV positive saliva among FEF with disseminated infection [20]. TE is the percentage of FEF with infectious saliva among all the FEF [21].

### Alphavirus, flavivirus and phlebovirus detection

As previous studies revealed arboviral circulation in the study area [22], female mosquitoes, which were subjected to artificial blood meals and intrathoracic inoculations, were screened by reverse transcription nested polymerase chain reactions (RT-nPCR) to detect phlebovirus (family *Phenuiviridae*) [23], flavivirus (family *Flaviviridae*) [24] and alphavirus (family *Togaviridae*) [25] natural infections. Amplified flavivirus NS5 gene fragments were purified, sequenced and submitted to a basic local alignment search tool (BLAST) query for taxonomic assignation. To discard a virus insertion in the mosquito genome, DNA extracts from the samples that tested positive for flavivirus were treated with Ribonuclease A (RNase A) (Sigma-Aldrich, St. Louis, MO, USA) [26] prior flavivirus PCR amplification.

### Statistics

In order to assess whether the natural infection of the Mosquito flavivirus influenced the vector competence for RVFV, the proportions of RVFV-infected mosquitoes in both Mosquito flavivirus-positive and Mosquito flavivirus-negative groups were compared using the Fisherʼs exact test [27]. Furthermore, we evaluated the differences in the mean RVFV Cq values of infected specimens depending on the presence/absence of the Mosquito flavivirus with a Wilcoxon test. All statistical analyses were carried out using R statistical software (http://cran.r-project.org/).

## Results

### *Aedes vexans* feeding and mortality rates

Four hundred and twenty-two and 148 *Ae. vexans* females emerged from field-collected larvae in 2016 and 2019, respectively. Low feeding rates (FR) were obtained after artificial blood meals [FR1 = 6.4% (27/422); FR2 = 12.6% (50/395); FR3 = 19.6% (29/148)].

In 2016, a mortality rate of 3.9% (3/77) was observed after blood-feeding; two and one deceased specimens exposed to RVFV and negative control groups, respectively. Meanwhile, in 2019 the mortality rates observed were 13.8% (4/29) and 21% (17/81) in females, which were orally exposed to RVFV and females subjected to intrathoracic inoculations, respectively.

### Flavivirus detection in the field-collected *Aedes vexans* population

In 2016, flavivirus RT-nPCR showed a 58.4% (45/77) natural infection with a Mosquito flavivirus (71-nucleotide fragment; 99% similarity with OcFV137A_09, GenBank: JN257977.1). A similar prevalence of the Mosquito flavivirus (53.9%; 48/89) was observed for this mosquito population in 2019. Ribonuclease A (RNase A)-treated DNA extracts were negative for flavivirus by RT-nPCR discarding viral genome insertions. Alphavirus and phlebovirus screening excluded natural infection in the studied *Ae. vexans* population.

### Vector competence of *Aedes vexans* for Rift Valley fever phlebovirus after oral exposure

**Fig. 1:**
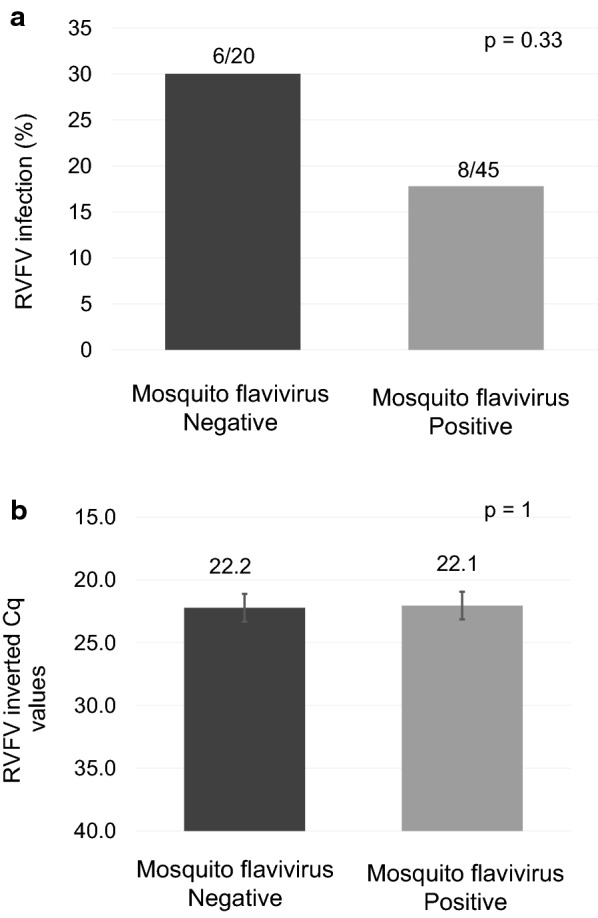
RVFV infection of *Aedes vexans* in relation to the presence or absence of a Mosquito flavivirus natural infection. **a** The proportion of RVFV infection is lower in mosquitoes naturally infected with a Mosquito flavivirus than in naturally non-infected mosquitoes. **b** RVFV mean Cq values in female bodies did not differ significantly in both groups, Mosquito flavivirus infected and non-infected. RVFV loads were not influenced by the Mosquito flavivirus infection

Vector competence estimators evidenced that the RVFV infectious dose used in the present study (7.5 log_10_ TCID_50_/ml in infectious blood) allowed the virus to infect the body, disseminate through the haemolymph and be transmitted by field-captured *Ae. vexans* mosquitoes (Tables 1, 2). Only 17.7% (8/45) of the mosquitoes naturally infected with flavivirus resulted in infection with RVFV in contrast to 30% (6/20) of non-flavivirus-infected mosquitoes (Table 1). However, given the small sample size, differences were not significant (*P* = 0.33). Additionally, no difference (*P* = 1) in the mean RVFV Cq values of infected specimens was observed between groups, with and without Mosquito flavivirus (Fig. 1).

**Table 1:** Vector competence of *Aedes vexans* for Rift Valley fever phlebovirus at 14 dpe. Infection, dissemination and transmission rates, and transmission efficiency of a natural *Ae. vexans* population from Catalonia, Spain orally exposed to Rift valley fever phlebovirus (RVFV 56/74)

Mosquito flavivirus infection status	Feeding 1					Feeding 2					Feeding 3				
	*n*	IR	DIR	TR	TE	*n*	IR	DIR	TR	TE	*n*	IR	DIR	TR	TE
Mosquito flavivirus-negative (%)	4	0/4 (0)	0/0 (0)	0/0 (0)	0/4 (0)	11	5/11 (45.0)	1/5 (20.0)	1/1 (100)	1/11 (9.1)	5	1/5 (20.0)	1/1 (100)	1/1 (100)	1/5 (20)
Mosquito flavivirus-positive (%)	12	1/12 (8.3)	1/1 (100)	0/1 (0)	0/12 (0)	23	3/23 (13.0)	1/3 (33.3)	1/1 (100)	1/23 (4.3)	10	4/10 (40.0)	2/4 (50)	2/2 (100)	2/10 (20)
Total (%)	16	1/16 (6.3)	1/1 (100)	0/1 (0)	0/16 (0)	34	8/34 (23.5)	2/8 (25.0)	2/2 (100)	2/34 (5.8)	15	5/15 (33.3)	3/5 (60)	3/3 (100)	3/15 (20)

*Notes*: IR, positive bodies/total fully engorged females; DIR, positive legs and wings/positive bodies; TR, positive saliva/ positive legs and wings; TE, positive saliva/total fully engorged females

*Abbreviations*: n, total fully engorged females; IR, infection rate; DIR, disseminated infection rate; TR, transmission rate; TE, transmission efficiency

**Table 2:** Relevance of the midgut and salivary glands barriers in *Aedes vexans* after oral exposure to RVFV 56/74

	IR MIB	DIR MEB	TR SB	TE	Overall vector competence
Relative importance (%)	14/65 (21.5) +++	6/14 (42.9) ++	5/6 (83.3) null^a^	5/65 (7.7)	Low

^a^Uncertain given the small sample size

*Notes*: Rating of relative importance of the barrier: null, virus crosses this barrier in >80% of females; +, minor, virus crosses this barrier in 60–80% of females; ++, moderate, virus crosses this barrier in 40–60% of females; +++, severe, virus crosses this barrier in 20–40% of females; ++++, very severe, virus crosses this barrier in < 20% of females [10]

*Abbreviations*: IR, infection rate; DIR, disseminated infection rate; TR, transmission rate; TE, transmission efficiency; MIB, midgut infection barrier; MEB, midgut escape barrier; SB, salivary gland barrier

Out of six specimens with disseminated infection, five tested positive for RVFV in saliva (TR of 83.3%) by RT-qPCR (Cq values: 22.38–33.94). The viability of RVFV viral particles of all these samples was confirmed by the cytopathic effect observed after incubation on Vero cell monolayers. Of the females which were able to transmit RVFV, three belonged to the Mosquito flavivirus naturally infected group; and two, to the non-infected group. For this *Ae. vexans* population, a transmission efficiency (TE) of 7.7% (5/65) was estimated.

### Evaluation of salivary gland barriers of *Aedes vexans* for Rift Valley fever phlebovirus after intrathoracic inoculation

At day seven post-inoculation (7 dpi), RVFV dissemination and infection in all the specimens subjected to intrathoracic inoculations were confirmed (DIR = 100%, 45/45 and IR = 100%, 45/45). All the saliva samples that tested positive for RVFV by RT-qPCR (37/45; Cq = 23.89–33.34) also showed cytopathic effect after incubation on Vero cell monolayers. An 82.2% transmission rate was estimated, out of 45 inoculated specimens, 37 were able to transmit the virus.

## Discussion

To our knowledge, the present study reports for the first time a European field population of *Ae. vexans* as a competent vector for RVFV. In our study, oral exposure to the virulent strain RVFV 56/74 (7.5 log_10_ TCID_50_/ml in infectious blood) denoted severe and moderate importance of the *Ae. vexans* midgut infection and escape barriers, respectively; the virus was unable to cross these barriers in 78.5% and 51.1% in the overall FEF in each case. Meanwhile, the salivary gland barriers seem to be less important when a disseminated infection has already occurred. In the present study, transmission rates after oral exposure to the virus (83.3%) and after intrathoracic microinjections (82.2%) indicate that once RVFV is circulating through the haemocoel it is capable of successfully infecting the salivary glands and can transmit through the mosquito saliva.

Our overall results suggest that the studied population of *Ae. vexans* exhibits a low vector competence for RVFV (TE of 7.7%). Similarly, a German *Ae. vexans* laboratory colony was categorized as a low competent vector when orally exposed to infectious blood meals containing the virulent ZH548 strain and the avirulent Clone 13 strain [28]. Previous studies have shown that *Ae. vexans* infection susceptibility and vector competence for RVFV is heterogeneous among geographically separated populations. In Senegal, for example, F1 specimens exposed to infectious blood meals containing three African strains (ArD141967, AnD133719 and SHM172805: at 4.5–9.5 × 10^6^ PFU), exhibited moderate significance of the MIB, MEB and salivary gland barriers (IR: 30–85%; DR: 10.5–37%; and TR: 13–33.3%) [9]. These results were in accordance with several studies conducted at the USA where field captured specimens were subjected to oral exposure to viraemic animals inoculated with a variety of ZH501 strain doses (10^4.1–10.2^PFU/ml) [10, 11]. In all cases, Senegalese and USA *Ae. vexans* populations showed a moderate RVFV vector competence. In contrast, studies that included populations from Canada [29], California and Colorado [30], where field *Ae. vexans* populations were exposed to highly viraemic animals, revealed an inability to disseminate and transmit RVFV, respectively. Divergent results, besides the mosquito populations, could also be explained by differences in the viral strains or the infection methodologies used in each case.

The finding that the autochthonous population of *Ae. vexans* studied was naturally infected with a field-circulating Mosquito flavivirus, and it was maintained in the field through the years, was an interesting outcome of the experiment. The prevalence of this Mosquito flavivirus was consistent in both sampling years. Regarding RVFV co-infection with the circulating Mosquito flavivirus, our results show that the presence of the Mosquito flavivirus seemed to decrease the susceptibility to RVFV infection, although this effect was not statistically significant. Contrasting results were observed in our previous study [31]. The vector competence of a *Culex pipiens* colony, which was previously infected intrathoracically with Culex Flavivirus (CxFV), for the same RVFV strain (RVFV 56/74) was not affected by the infection with the CxFV. Diverse outcomes have been observed in several co-infection studies involving an insect-specific virus and a pathogenic one. For instance, in Colorado, *Cx. pipiens* naturally infected with CxFV showed a possible suppression in West Nile virus (WNV) early infection [32]. A similar co-infection, in *Culex quinquefasciatus* from Honduras, had the opposite effect, an enhancement of WNV transmission [33]. *Aedes triseriatus* turned out to be resistant to Snowshoe hare virus infection in presence of LaCrosse virus, a closely related bunyavirus [34]. Further studies are required to clarify the potential role of the Mosquito flavivirus in the infection susceptibility and transmission of RVFV in the *Ae. vexans* population studied.

Finally, the experimental confirmation of a European biting nuisance species, such as *Ae. vexans*, as a RVFV vector highlights the necessity of regular and exhaustive arboviral vector surveillance and control strategies in susceptible areas in the Mediterranean region, where *Ae. vexans* is distributed, to avoid a possible outbreak in the case of RVFV introduction.

## Conclusions

The present study demonstrates for the first time that a European field-collected population of *Ae. vexans* may be involved in the transmission of RVFV in case of introduction to the continent. This knowledge contributes to the development of more accurate strategies for vector surveillance and control of RVF. The naturally circulating Mosquito flavivirus seems to modulate the susceptibility to RVFV infection in the assessed population of *Ae. vexans*. Further studies are needed to elucidate the potential of insect-specific viruses for the development of new biotools for the control of sanitary relevant arboviruses and their vectors.

## Data Availability

All data generated or analyzed during this study are included in this published article.

## Abbreviations

WNV: West Nile virus
SSHV: Snowshoe hare virus
JCV: Jamestone Canyon virus
TAHV: Tahyna virus
BATV: Batai virus
RVFV: Rift Valley fever phlebovirus
RVF: Rift Valley fever
OIE: World Organization for Animal Health
NIAID: National Institute of Allergy and Infectious Diseases
MCDM: multicriteria decision-making
FEF: fully engorged females
RT-nPCR: reverse transcription nested polymerase chain reactions
RNAse A: ribonuclease A
IR: infection rate
DIR: disseminated infection rate
TR: transmission rate
TE: transmission efficiency
Cq: quantification cycle
RT-qPCR: reverse transcription quantitative polymerase chain reaction
MIB: midgut infection barrier
MEB: midgut escape barrier
SB: salivary glands barrier
CxFV: Culex flavivirus
ATP: adenosine 5′-triphosphate
SGICE: Servei de granja i camps experimentals
TCID_50_/ml: 50% tissue culture infective dose per millilitre
IRTA - CReSA: Institut de Recerca i Tecnologies Agroalimentaries - Centre de Recerca en Sanitat Animal
BSL3: biosafety level 3
SPF: specific pathogen-free
EIP: extrinsic incubation period
dpe: days post-exposure
dpi: days post inoculation
DMEM: Dulbecco’s modified Eagle’s medium
BLAST: basic local alignment search tool
